# Effect of *Alpinia calcarata* on glucose uptake in diabetic rats-an in vitro and in vivo model

**DOI:** 10.1186/2251-6581-13-33

**Published:** 2014-02-06

**Authors:** Ramya Rajasekar, Kalaiselvi Manokaran, Narmadha Rajasekaran, Gomathi Duraisamy, Devaki Kanakasabapathi

**Affiliations:** 1 Department of Biochemistry, Karpagam University, Coimbatore 641 021, Tamilnadu, India; 2 PG & Research Department of Biotechnology, Kongunadu Arts and Science College, Coimbatore 641 029, Tamilnadu, India; 3 Department of Biochemistry, College of Medical Lab Technology, Acharya Institute of Health Science, Bangalore, India

**Keywords:** Diabetes mellitus, Streptozotocin, Glibanclamide, Hemidiaphragm

## Abstract

**Background:**

Diabetes mellitus is a heterogeneous metabolic disorders characterized by abnormally high levels of blood glucose The main objective of the present work is to study the effect of *Alpinia calcarata* on glucose uptake in streptozotocin (STZ) induced diabetic rats.

**Methods:**

The diabetes was induced by single dose of STZ (45 mg/kg) in citrate buffer, while the normal control group was given the vehicle (citrate buffer) only. After induction of diabetes, the diabetic animals were treated with ethanolic extract of *Alpinia calcarata* (200 mg/kg) and glibenclamide (2 mg/kg) for 30 days. Blood glucose estimation was performed every week of the study. At the end of study period, animals were sacrificed for biochemical studies.

**Results:**

Streptozotocin induced diabetic rats shows the altered levels of various biochemical profiles. Those levels were brought back to near normal upon treatment with ethanolic extract of *Alpinia calcarata* and standard drug glibanclamide. No significant changes were observed on treatment with plant extract alone group indicated that there are no toxic substances present in *Alpinia calcarata*. The antidiabetic activity of plant extract was also further confirmed by histopathological studies. The ethanolic extract of *Alpinia calcarata* shows significant inhibition of alpha glucosidase activity and also enhancing the glucose uptake in rat hemidiaphragm.

**Conclusions:**

In conclusion, the ethanolic extract of *Alpinia calcarata* ameliorates the condition associated with diabetes.

## Introduction

Diabetes mellitus is a heterogeneous metabolic disorders characterized by abnormally high levels of blood glucose. Diabetes mellitus has been recognized as a growing world-wide epidemic by many health’s advocacy groups including the World Health Organization (WHO). It is a major public health problem affecting 285 million or 6.4% of the world populations for the year 2010, reported by International Diabetes Federation 2009. By 2030, this number is estimated to rise to 435 million. Despite considerable progress in the management of diabetes mellitus using synthetic drugs, the search for improved and safe natural anti-diabetic agents with minimal side effects is ongoing [1].

The current treatment for control of diabetes mellitus includes diet, exercise, oral anti diabetic drugs and insulin therapy. However, insulin and other oral hypoglycemic drugs have characteristic profile of adverse effects. This has initiated the identification of novel drugs which might act in mechanistically distinct way compared to existing drug targets [2]. Hence, research is focused on medicinal plants which are used in the practices and development of newer drug leads from phytoconstituents with more potential and effective agents with lesser side effects than the existing hypoglycemic agents [3]. Many medicinal plants are currently used in India for the treatment of diabetes and scientifically its efficacy has been proved earlier [4].

*Alpinia calcarata* is one such medicinal plant which belonging to the family Zingiberaceae. It is cultivated in tropical countries including India, Sri Lanka and Malaysia. The plants are widely used to relieving stomachache, treating colds, invigorating the circulatory system and reducing swellings. Experimentally, rhizomes of *Alpinia calcarata* are shown to possess antibacterial, antifungal, anthelmintic, antinociceptive, antioxidant, aphrodisiac and gastroprotective activities. The rhizomes of *Alpinia calcarata* is considered as aphrodisiac and used in the treatment of arthritis, bronchitis, cough, respiratory ailments, asthma and diabetes [5]. The present study was conducted to evaluate the effect of *Alpinia calcarata* on glucose uptake in streptozotocin (STZ) induced diabetic rats.

## Materials and methods

### Collection of plant material

The rhizome of *Alpinia calcarata* were procured from local place, identified and authentified by Dr. G.V.S. Murthy, Botanical Survey of India, TamilNadu Agricultural University (TNAU), Coimbatore (Voucher no: BSI/SRC/5/23/09-10/Tech-982).

### Preparation of solvent extraction

About 50 g of powdered plant material was mixed with 250 ml of ethanol in a 500 ml conical flask and was placed in a shaker for 16 hours. The solution was then extracted using a separating funnel and was concentrated by solvent evaporation using rotary evaporator. The sample material was stored in an air tight container at 4°C and used for further experimental analysis.

### Experimental animals

Adult albino rats weighing about 150–200 g were obtained from the animal house of Karpagam University, Coimbatore and were used for the study. Rats were housed in polycarbonate cages in a room with a 12-hour day-night cycle, at constant temperature of 22°C and humidity of 45-64%. During the experimental study, the rats were fed on pellets (Gulmohur rat feed, Lipton India, Bangalore) with free access to tap water. The rats received humane care according to the criteria outlined in Principles of Laboratory animal care, 1985.

All the experiments were carried out according to the guidelines recommended by the Committee for the Purpose of Control and Supervision of Experiments on Animals (CPCSEA) and approved by Institutional Animal Ethics Committee (IAEC), Government of India for the use of rats as an *in vivo* and *in vitro* animal model for diabetic studies [6].

### Experimental pattern

The animals were divided into five groups of six animals each. Group 1 served as control animals given normal pelleted diet and 1.0 ml citrate buffer as vehicle, group 2 rats were induced with single intraperitoneal injection of freshly prepared streptozotocin (45 mg/kg body weight) in 0.1 M citrate buffer (pH 4.5) in a volume of 1 ml/kg body weight, group 3 rats were induced with diabetes and treatment with standard drug glibenclamide (2 mg/kg body weight) through oral intragastric tube for 30 days, group 4 animals were induced with diabetes and treatment with ethanolic extract of *Alpinia calcarata* (200 mg/kg body weight) orally for 30 days and group 5 control rats were treated with *Alpinia calcarata* only for 30 days at a concentration of 200 mg/kg body weight.

Blood glucose levels were measured in normal and experimental rats at initial, 15^th^ and 30^th^ days of treatment using electronic glucometer and the experiment was terminated in overnight fasted rats at the end of 30 days.

### Sacrification of animals

After experimental period, rats were sacrificed by cervical dislocation after giving mild anesthesia using chloroform. Blood was collected and the serum was separated by centrifugation at 20000 rpm for 30 minutes. Liver and Kidney were immediately dissected out, washed and stored in 0.9% ice cold saline for various biochemical evaluations and tissues stored in 1% formalin were used for histopathological studies. Diaphragm was used to analyze the glucose uptake assay and small intestine was used to determined inhibition of α-glycosidase activity.

### Estimation of biochemical profiles

The serum was used for the analysis of glucose [7], cholesterol, triglycerides, HDL cholesterol was estimated by one step method using diagnostic reagent kit manufactured by Span diagnostics Ltd, VLDL was calculated by the formulae: VLDL (mg/dl) = Triglycerides/5, LDL was calculated by the formulae: LDL (mg/dl) = cholesterol – HDL -VLDL, urea was estimated by DAM method using diagnostic reagent kit manufactured by Span diagnostics Ltd, creatinine was estimated by alkaline picrate method using a reagent kit intended for in vitro quantitative determination manufactured by Beacon diagnostics Pvt Ltd, AST, ALT and ALP was estimated by method using diagnostic reagent kit manufactured by Span diagnostics Ltd.

Tissue homogenates was used to analyze the enzymatic and non-enzymatic antioxidant and the parameters analyzed were Protein was estimated by Lowry’s et al., [8], superoxide dismutase were determined according to the method of Misra and Fridovich [9], catalase by Sinha [10], glutathione peroxidase by Rotruck et al., [11], glutathione-s- transferase by Habig et al., [12], glutathione by Moron et al., [13], vitamin c by Omaye et al., [14], basal lipid peroxidation by Hogberg et al., [15], ascorbate induced lipid peroxidation was determined by Devasagayam and Tarachand [16], peroxide induced lipid peroxidation was estimated according to the method given by Devasagayam and Tarachand [17].

Estimation of glucose uptake by isolated rat hemidiaphragm was carried out by the method of Chattopadhyay et al., [18] and inhibition assay for the α-glucosidase activity was done by the method of Dahlqvist [19].

### Statistical analysis

All the data were expressed as Mean ± S.D. Statistical significance was evaluated by one way analysis of variance (ANOVA) using SPSS version 7.5 (SPSS, Cary, USA) and the individual comparisons were obtained by the Duncan’s Multiple Range Test (DMRT). A value of p < 0.05 was considered to indicate a significant difference between graphs.

## Results and discussion

Diabetes mellitus patients in India are increasing day by day probably due to change in lifestyle change in food pattern i.e. from traditional fiber rich diet to sugary fast food diet and also because of genetic basis. The disorder being chronic in nature needs long term treatment to prevent the complications arising due to persistent high blood glucose level. Pharmacotherapy available for the treatment of diabetes in modern healthcare system includes insulin and oral hypoglycemic drugs. However due to economic constraints, it is not possible for majority of the diabetic patients in developing countries like India to use these drugs on regular basis [20].

Moreover the synthetic antidiabetic drugs are associated with large number of adverse effects. Hence there is increase in the trend to use traditional indigenous plants widely available in India for the treatment of diabetes mellitus. The effective components of medicinal plants that have antidiabetic property include alkaloids, oligosaccharides, polysaccharides, organic acids and flavonoids etc. Streptozotocin (STZ) induced diabetes has been described as a useful experimental model to study the activity of antidiabetic agents [21]. In the present study the activity of ethanolic extract of *Alpinia calcarata* rhizome was evaluated in streptozotocin induced diabetic rats.

**Figure 1:**
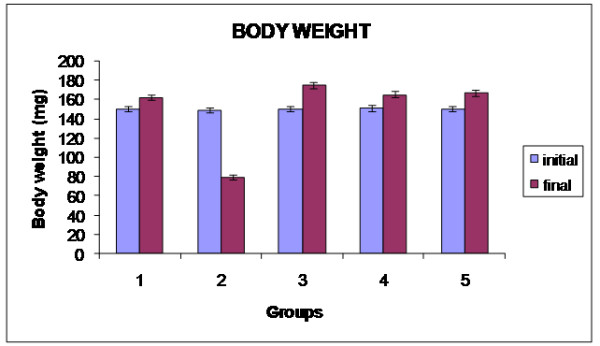
Changes in body weight in experimental rats.

The effect of the ethanolic extract *Alpinia calcarata* on body weight in the STZ induced diabetic rats are shown in Figure 1, the results indicate that the body weight of the untreated diabetic rats was found to be significantly decreased when compared with the normal control group. The ethanolic extract of *Alpinia calcarata* ameliorated this weight loss in STZ induced diabetic rats and from this it is evident that the extract possesses a significant beneficial effect. In diabetes mellitus, body cells are unable to utilize glucose as a source of energy due to which proteins are spared as energy source. This leads to decrease in protein storage which in turn reduces body weight. In the present study streptozotocin diabetic rats show decrease in body weight throughout the experimental period [22]. Oral treatment with aqueous extract of *Alpinia calcarata* significantly improved the body weight loss in diabetic rats as compared to diabetic control indicating possible role of the extract in restoration of protein metabolism. Hence, the weight gain after administration of the extract in severely diabetic rats is simply due to the ability of the extract to reduce diabetes [23].

Levels of blood glucose in experimental rats were shown in Table 1. Streptozotocin (STZ) diabetic rats exhibited significant increase in blood glucose level.

**Table 1:** Levels of blood glucose at various stages of study

**Grouping/days**	**0th Day (a)**	**15th Day (b)**	**30th Day (c)**
**Control**	110.46 ± 0.403^CNS^	112.46 ± 0.403^CNS^	111.5 ± 0.357^CNS^
**Diabetic control**	298.33 ± 0.314^**^	398.66 ± 0.372^**^	501.7 ± 0.322^**^
**Diabetic + Glibenclamide**	300.5 ± 0.357^**^	256.46 ± 0.36^**^	121.4 ±0.403^**^
**Diabetic +** ***Alpinia calcarata***	295.53 ± 0.361^NS^	270.4 ± 0.357^NS^	130.4 ± 0.314^**^
***Alpinia calcarata*** **alone**	109 ± 0.403^NS^	110.46 ± 0.314^NS^	110.5 ± 0.361^NS^

Values are expressed as Mean ± S.D.

Values are taken as a mean of five individuals’ experiment.

a = 0^th^ day compared with 15th day.

b = 15^th^ day compared with 30th day.

c = 0^th^ day compared with 30th day.

NS – Non Significant **-p < 0.05.

Upon treatment with ethanolic extract of *Alpinia calcarata* the blood glucose level was reduced throughout the experimental period in duration dependent manner indicating its antihyperglycemic activity. However blood glucose levels were not altered in normoglycemic rats further strengthening the antidiabetogenic potential of the extract.

Metabolic disturbances of carbohydrates, lipids and proteins during diabetes mellitus cause insulin deficiency stimulating lipolysis in adipose tissues, therefore this leads to fatty liver, hypercholesterolemia and hypertriglyceridemia. Furthermore increased triglycerides result in increase in free fatty acid level and its oxidation disturbs metabolism as well as utilization of glucose and also impairs insulin action leading to development of hyperglycemia [24]. The levels of lipid and lipoprotein profiles such as cholesterol, triglycerides, HDL, VLDL and LDL cholesterol in serum of experimental animals were depicted in Table 2. The levels of cholesterol, triglyceride, VLDL and LDL in serum were increased (p < 0.05) significantly whereas the level of lipoprotein lipase and HDL was decreased significantly (p < 0.05) in STZ induced animals when compared to control animals. Upon administration of *Alpinia calcarata* extract and glibenclamide the levels were significantly reverted back to the near normal levels when compared to normal animals. No significant changes were observed in plant extract alone treated animals when compared to control animals.

**Table 2:** Changes in levels of lipid and lipoprotein profiles in serum of experimental animals

**Groups**	**Cholesterol**	**Triglycerides**	**HDL**	**LDL**	**VLDL**
	**(mg/dl)**	**(mg/dl)**	**(mg/dl)**	**(mg/dl)**	**(mg/dl)**
**Control**	122.7 ± 0.32^a^	67.93 ± 0.36^a^	24.5 ± 0.31^a^	84.7 ± 0.32^a^	13.5 ± 0.31^a^
**Diabetic control**	204.4 ± 0.35^b^	221.9 ± 0.31^b^	13.4 ± 0.31^b^	146.5 ± 0.31^b^	44.5 ± 0.35^b^
**Diabetic + Glibenclamide**	112.9 ± 0.36^c^	71 ± 0.35^c^	23.16 ± 0.40^c^	75.5 ± 0.36^a^	14.23 ± 0.40^ac^
**Diabetic +** ***Alpinia calcarata***	124.23 ± 0.31^ad^	89.43 ± 0.31^d^	30.13 ± 0.36 ^d^	72.23 ± 0.40^d^	21.73 ± 0.36^d^
***Alpinia calcarata*** **alone**	106.3 ± 0.26^e^	73.46 ± 0.36^e^	31.33 ± 0.31^e^	70.6 ± 0.40^e^	14.4 ± 0.32^e^

Values are expressed as Mean ± S.D. Values are taken as a mean of five individuals experiments.

Values not sharing a common superscript letter differ significantly (DMRT).

^a^ Control group.

^b^ Comparison between control and diabetic groups.

^c^ Comparison between diabetic and standard drug glibenclamide treated groups.

^d^ Comparison between diabetic and *Alpinia calcarata* treated groups.

^e^ Comparison between control and *Alpinia calcarata* alone treated groups.

The present study showed increase in serum triglycerides, total cholesterol and LDL cholesterol with decrease in HDL cholesterol supporting the findings of the other researchers [23]. Potential of the extract to decrease cholesterol and triglyceride levels could be helpful in improving lipid metabolism in diabetics which in turn will help to prevent diabetic complications. LDL- cholesterol being involved in the transport of cholesterol from liver to peripheral tissues is the key factor in atherogenesis. Potential of the extract to reduce LDL-cholesterol thereby indicates its possible involvement in the prevention of diabetes mellitus induced cardiovascular complications [25].

The activities of AST, ALT and ALP in serum of experimental animals were shown in Table 3. The activities of these enzymes were found to be significantly increased (p < 0.05) in diabetes induced animals than in control animals. On treatment with plant extract, the levels were significantly decreased in a dose dependent manner showing a favourable change in groups treated with 200 mg/kg. Treatment with ethanolic extract alone did not show any significant difference when compared to normal control group.

**Table 3:** Levels of hepatic marker enzymes in serum of experimental animals

**Groups**	**SGOT (IU/L)**	**SGPT (IU/L)**	**ALP (IU/L)**
**Control**	122.03 ± 0.314^a^	83.1 ± 0.322^a^	259.9 ± 0.314^a^
**Diabetic control**	272.93 ± 0.361^b^	191.8 ± 0.372^b^	381.8 ± 0.422^b^
**Diabetic + Glibenclamide**	200.9 ± 0.409^c^	80.96 ± 0.314^c^	295 ± 0.357^c^
**Diabetic +** ***Alpinia calcarata***	230.1 ± 0.322^d^	79.36 ± 0.361^d^	241.9 ± 0.361^d^
***Alpinia calcarata*** **alone**	120.03 ± 0.492^ae^	83.5 ± 0.389^a^	254.9 ± 0.409^e^

Values are expressed as Mean ± S.D. Values are taken as a mean of five individuals’ experiments.

Values not sharing a common superscript letter differ significantly (DMRT).

^a^ Control group.

^b^ Comparison between control and diabetic groups.

^c^ Comparison between diabetic and standard drug glibenclamide treated groups.

^d^ Comparison between diabetic and *Alpinia calcarata* treated groups.

^e^ Comparison between control and *Alpinia calcarata* alone treated groups.

The measurement of the activities of marker or diagnostic enzymes in serum plays a significant and well-known role in diagnosis, disease investigation and in the assessment of drug or plant extract for safety or toxicity risk. The enzymes considered in this study are useful marker enzymes of liver cytolysis and damage to the plasma membrane of the liver cells. In the present study shows an increased in the level of serum marker enzymes in STZ induced diabetes indicating that STZ administration produced hepatic damage [26].

The levels of urea and creatinine were shown in Table 4. The levels of urea and creatinine were significantly increased in group II diabetic induced animals. These levels were reverted back in extract and glibenclamide treated animals. Whereas, the group V plant extract alone rats the above levels were similar to the control groups. Kidney maintains optimum chemical composition of body fluid by acidification of urine and removal of metabolic wastes such as urea, uric acid, creatinine and ions. During renal diseases, the concentration of these metabolites increases in blood [27]. In the present study it was observed that, administration of plant extract at 200 mg/kg doses reduced elevated levels of urea and creatinine, which was comparable to the effect observed with glibenclamide. This indicates the prevention of kidney damage, which may be possible in diabetic animals [28].

**Table 4:** Levels of renal markers in serum of experimental animals

**Groups**	**Urea (mg/dl)**	**Creatinine (mg/dl)**
**Control**	19.3 ± 0.357^a^	0.75 ± 0.040^a^
**Diabetic control**	42.5 ± 0.357^b^	2.53 ± 0.049^b^
**Diabetic + Glibenclamide**	22.53 ± 0.361^c^	0.74 ± 0.053^a^
**Diabetic +** ***Alpinia calcarata***	22.53 ± 0.361^c^	1.66 ±0.372^c^
***Alpinia calcarata*** **alone**	22.2 ± 0.389^c^	0.66 ± 0.322^d^

Values are expressed as Mean ± S.D. Values are taken as a mean of five individuals’ experiments.

Values not sharing a common superscript letter differ significantly (DMRT).

^a^ Control group.

^b^ Comparison between control and diabetic groups.

^c^ Comparison between diabetic and standard drug glibenclamide treated groups.

^d^ Comparison between control and *Alpinia calcarata* alone treated groups.

Tables 5 and 6 shows the effect of ethanolic extract of *Alpinia calcarata*. The results showed that the activity of catalase, superoxide dismutase, glutathione peroxidase and glutathione-S-transferase decreased significantly in STZ induced diabetic group compared to normal control group. Ethanolic extract of *Alpinia calcarata* treated diabetic rats is significantly increased the antioxidant enzyme activities and reversed them to their near normal levels. The same phenomenon was seen in the results of glibenclamide treated groups. Normal rats treated with ethanolic extract of *Alpinia calcarata* showed the same effect as that of control rats and there was no significant change.

**Table 5:** Levels of enzymatic antioxidants in liver of experimental animals

**Groups**	**Superoxide dismutase**	**Catalase**	**Glutathione-S-transferase**	**Glutathione peroxidase**
**Control**	7.46 ± 0.40 ^a^	63.06 ± 0.361^a^	9.07 ± 0.054^a^	28.11 ± 0.040^a^
**Diabetic control**	4.2 ± 0.357^b^	42.01 ± 0.040^b^	4.82 ± 0.040^b^	11.43 ± 0.441^b^
**Diabetic + Glibenclamide**	6.90 ± 0.045^c^	60.11 ± 0.03^c^	8.77 ± 0.054^c^	23.06 ± 0.049^c^
**Diabetic +** ***Alpinia calcarata***	6.53 ± 0.044^cd^	58.77 ± 0.049^d^	8.61 ± 0.031^d^	26.64 ± 0.045^d^
***Alpinia calcarata*** **alone**	7.21 ± 0.049^a^	64.24 ± 0.044^ae^	8.97 ± 0.049^ae^	27.25 ± 0.036^e^

Values are expressed as Mean ± S.D. Values are taken as a mean of five individuals experiments.

Values not sharing a common superscript letter differ significantly (DMRT).

Units:

Superoxide dismutase- Units/mg of protein.

Catalase- (μ moles of H_2_O_2_ utilized /min/mg/protein.

Glutathione-S-transferase- μmoles of CDNB-Conjugate formed/mg of protein.

Glutathione peroxidase- μg of GSH/mg of protein.

^a^ Control group.

^b^ Comparison between control and diabetic groups.

^c^ Comparison between diabetic and standard drug glibenclamide treated groups.

^d^ Comparison between diabetic and *Alpinia calcarata* treated groups.

^e^ Comparison between control and *Alpinia calcarata* alone treated groups.

**Table 6:** Levels of enzymatic antioxidants in kidney of experimental animals

**Groups**	**Superoxide dismutase**	**Catalase**	**Glutathione-S-transferase**	**Glutathione peroxidase**
**Control**	1.5 ± 0.357^a^	26.76 ± 0.546^a^	7.03 ± 0.031^a^	31.12 ± 0.040^a^
**Diabetic control**	0.86 ± 0.044^b^	18.76 ± 0.045^b^	3.46 ± 0.040^b^	14.17 ± 0.049^b^
**Diabetic + Glibenclamide**	1.24 ± 0.040^ac^	25.16 ± 0.049^c^	6.73 ± 0.361a^c^	24.1 ± 0.044^c^
**Diabetic +** ***Alpinia calcarata***	1.03 ± 0.037^bc^	19.66 ± 0.044^d^	6.52 ± 0.045^c^	22.83 ± 0.035^d^
***Alpinia calcarata*** **alone**	1.37 ± 0.042^ac^	26.37 ± 0.040^a^	6.96 ± 0.040 ^a^	30.12 ± 0.036^a^

Values are expressed as Mean ± S.D. Values are taken as a mean of five individuals experiments.

Values not sharing a common superscript letter differ significantly (DMRT).

Units:

Superoxide dismutase- Units/mg of protein.

Catalase- (μ moles of H_2_O_2_ utilized /min/mg/protein.

Glutathione-S-transferase- μmoles of CDNB-Conjugate formed/mg of protein.

Glutathione peroxidase- μg of GSH/mg of protein.

^a^ Control group.

^b^ Comparison between control and diabetic groups.

^c^ Comparison between diabetic and standard drug glibenclamide treated groups.

^d^ Comparison between control and *Alpinia* calcarata alone treated groups.

An imbalance between the production and scavenging of free radicals can result in increased oxidative stress. Increased free radical generation and oxidative stress are hypothesized to play an important role in the pathogenesis of diabetes and its later complications. Diabetic state is shown to be associated with depletion of antioxidants [29]. Administration of *Alpinia calcarata* showed a protective effect against free radical damage.

In liver and kidney the concentration of vitamin C and glutathione was decreased significantly in untreated diabetic rats, when compared to the normal groups. On treatment with *Alpinia calcarata*, significant increase in the levels of non-enzymatic antioxidants when compared with untreated diabetic rats were observed. There was no change in the level of the above parameters in the rats which were given the plant extract alone and is almost similar to control group (Tables 7 and 8). Antioxidants exist in interconvertable (reduced and oxidized) forms. The decreased level of ascorbic acid in diabetic rats may be due to either increased utilization as an antioxidant defense against increased ROS or to a decrease in glutathione level since glutathione is required for the recycling of ascorbic acid [30].

**Table 7:** Levels of non-enzymatic antioxidants in liver of experimental animals

**Groups**	**Total reduced glutathione**	**Vitamin C**
	**(μg/mg protein)**	**(μg/mg protein)**
**Control**	47.47 ± 0.049^a^	1.46 ± 0.040^a^
**Diabetic control**	28.95 ± 0.040^b^	0.72 ± 0.044^b^
**Diabetic + Glibenclamide**	43.53 ± 0.045^c^	1.37 ± 0.049^cd^
**Diabetic +** ***Alpinia calcarata***	43.22 ± 0.033^d^	1.36 ± 0.045^d^
***Alpinia calcarata*** **alone**	46.9 ± 0.035^a,e^	1.42 ± 0.035^a,d^

Values are expressed as Mean ± S.D. Values are taken as a mean of five individuals experiments.

Values not sharing a common superscript letter differ significantly (DMRT).

^a^ Control group.

^b^ Comparison between control and diabetic groups.

^c^ Comparison between diabetic and standard drug glibenclamide treated groups.

^d^ Comparison between diabetic and *Alpinia calcarata* treated groups.

^e^ Comparison between control and *Alpinia calcarata* alone treated groups.

**Table 8:** Levels of non-enzymatic antioxidants in kidney of experimental animals

**Groups**	**Total reduced glutathione**	**Vitamin C**
	**(μg/mg protein)**	**(μg/mg protein)**
**Control**	45.76 ± 0.049^a^	1.20 ± 0.049^a^
**Diabetic control**	20.9 ± 0.357^b^	0.44 ± 0.040^b^
**Diabetic + Glibenclamide**	38.41 ± 0.040^c^	1.11 ± 0.044^a^
**Diabetic +** ***Alpinia calcarata***	33.33 ± 0.314^d^	1.04 ± 0.035^a^
***Alpinia calcarata*** **alone**	45.64 ± 0.045^a^	1.14 ± 0.045^a^

Values are expressed as Mean ± S.D. Values are taken as a mean of five individual experiments.

Values not sharing a common superscript letter differ significantly (DMRT).

^a^ Control group.

^b^ Comparison between control and diabetic groups.

^c^ Comparison between diabetic and standard drug glibenclamide treated groups.

^d^ Comparison between control and *Alpinia* calcarata alone treated groups.

Lipid peroxidation is a free radical mediated process leading to oxidative deterioration of polyunsaturated lipids. Under normal physiological conditions, low concentrations of lipid peroxides are found in plasma and tissues. Oxygen derived free radicals generated in excess in response to various stimuli could be cytotoxic to several tissues. Most of the tissue damage is considered to be mediated by these free radicals by attacking membranes through peroxidation of polyunsaturated fatty acids. The increase in oxygen free radicals in diabetes could be primarily due to increase in blood glucose levels, which upon auto-oxidation generate free radicals [31].

The effect of the ethanolic extract of *Alpinia calcarata* on the liver and kidney lipid peroxidation is given in Table 9. The elevated levels of basal lipid peroxidation, ascorbate induced lipid peroxidation and peroxide induced lipid peroxidation in streptozotocin induced diabetic rats was reduced significantly to near normal levels upon treatment with *Alpinia calcarata* extract and the standard drug glibenclamide. The plant extract alone treated rats did not show any significant change. The increased susceptibility of the tissues of the diabetic animals may be due to the activation of the lipid peroxidation system. The possible source of oxidative stress in diabetes includes shifts in redox balance resulting from altered carbohydrate and lipid metabolism, increased generation of reactive oxygen species [32].

**Table 9:** Levels of lipid peroxidation in liver of experimental animals

**Groups**	**Lipid peroxidation**	**Ascorbate induced lipid peroxidation**	**Peroxide induced lipid peroxidation**
**Control**	11.05 ± 0.04^a^	13.76 ± 0.04^a^	9.93 ± 0.36^a^
**Diabetic control**	25.9 ± 0.32^b^	32.34 ± 0.03^b^	23.34 ± 0.04^b^
**Diabetic + Glibenclamide**	14.76 ± 0.03^c^	18.3 ± 0.04^c^	13.33 ± 0.04^c^
**Diabetic +** ***Alpinia calcarata***	15.9 ± 0.04^d^	19.88 ± 0.04^d^	14.45 ± 0.04^d^
***Alpinia calcarata*** **alone**	11.4 ± 0.04^a^	14.22 ± 0.05^e^	10.33 ± 0.03^e^

Values are expressed as Mean ± S.D. Values are taken as a mean of five individual experiments.

Values not sharing a common superscript letter differ significantly (DMRT).

Units:

Lipid peroxidation- nmoles of MDA formed/g tissue.

^a^ Control group.

^b^ Comparison between control and diabetic groups.

^c^ Comparison between diabetic and standard drug glibenclamide treated groups.

^d^ Comparison between diabetic and *Alpinia calcarata* treated groups.

^e^ Comparison between control and *Alpinia calcarata* alone treated groups.

Glucose uptake increased significantly with insulin alone treated rats and rhizome of *Alpinia calcarata* alone treated rats, but the uptake is more in insulin treated group than the rhizome treated group. Treatment of rats with both insulin and *Alpinia calcarata* increase the glucose uptake very significantly. In *Alpinia calcarata* alone treated group increased the glucose uptake in rat hemidiaphragm which was higher than that of insulin treated diaphragms (Table 10). The enhanced glucose utilization of hemidiaphragm in presence of rhizome extract revealed that the glucose uptake is similar to that of insulin. These findings suggest that the ethanolic extract of *Alpinia calcarata* may have direct insulin like activity which enhances the peripheral utilization of glucose and have extra pancreatic effect [26].

**Table 10:** **Effect of *Alpinia calcarata *** **on glucose uptake in isolated rat hemidiaphragm- in vitro assay**

**Particulars**	**Control**	**Diabetic control**	**Diabetic + Glibenclamide**	**Diabetic +** ***Alpinia calcarata***	***Alpinia calcarata*** **alone**
Tyrode solution with glucose (2 g%)	6.02 ± 0.10^a^	5.0 ± 0.08^b^	7.0 ± 0.12^c^	9.04 ± 0.10^d^	8.0 ± 0.10^e^
Tyrode solution with glucose (2 g%) + insulin (0.25 IUmL^-1^ )	10.02 ± 0.12^a^	11.04 ± 0.09^b^	11.99 ± 0.10^c^	13.0 ± 0.12^d^	9.01 ± 0.10^e^
Tyrode solution with glucose (2 g%) + Rhizome extract (200 mg L^-1^)	8.01 ± 0.06^a^	9.02 ± 0.08^b^	6.99 ± 0.10^c^	10.02 ± 0.10^d^	12.03 ± 0.11^e^
Tyrode solution with glucose (2 g%) + insulin (0.25 IUmL ^1^) + Rhizome extract (200 mg mL^-1^)	12.01 ± 0.10^a^	14.02 ± 0.10^b^	14.02 ± 0.11^b^	13.00 ± 0.09^c^	11.0 ± 0.12^d^

Values are expressed as Mean ± S.D. Values are taken as a mean of five individual experiments.

Values not sharing a common superscript letter differ significantly (DMRT).

^a^ Control group.

^b^ Comparison between control and diabetic groups.

^c^ Comparison between diabetic and standard drug glibenclamide treated groups.

^d^ Comparison between diabetic and *Alpinia calcarata* treated groups.

^e^ Comparison between control and *Alpinia calcarata* alone treated groups.

*In vitro* α-glucosidase inhibition of the ethanolic extract of *Alpinia calcarata* treated rats showed a significant inhibitory action of alpha glucosidase enzyme (Table 11). The results revealed that plant extract showed 6.2% of inhibition at 50 μg/ml and 59.4% of inhibition at 500 μg/ml. There was a proportionate increase in the percentage of α- glucosidase inhibition in a concentration dependent manner. Acarbose was used as a reference standard for the evaluation of α- glucosidase inhibitory action.

**Table 11:** **In vitro alpha glucosidase inhibition using *Alpinia calcarata***

**Concentration**	**% inhibition of α-glucosidase activity**	
**(μg/ml)**	**Ethanolic extract of** ***Alpinia calcarata***	**Acarbose**
50	6.2 ± 0.70	19.22 ± 0.03
100	19.2 ± 0.86	35.43 ± 0.01
200	29.2 ± 1.89	50.03 ± 0.02
400	45.7 ± 1.80	67.58 ± 0.03
500	59.4 ± 1.78	88.12 ± 0.01
IC_50_ (μg/ml)	435 ± 3.57	200 ± 4.03

Alpha glucosidase are the enzymes involved in the metabolism of carbohydrates. Alpha amylase degrades complex dietary carbohydrates to oligosaccharides and disaccharides, which are ultimately converted into monosaccharide by alpha glucosidase. Liberated glucose is then absorbed by the gut and results in postprandial hyperglycemia. Inhibition of alpha glucosidase limits postprandial glucose levels by delaying the process of carbohydrate hydrolysis and absorption. The plant based alpha glucosidase inhibitor offers a prospective therapeutic approach for the management of post-prandial hyperglycemia [33]. In the present study, *Alpinia calcarata* exhibited appreciable alpha glucosidase inhibitory effects when compared with standard drug acarbose.

### Histopathological study

**Figure 2:**
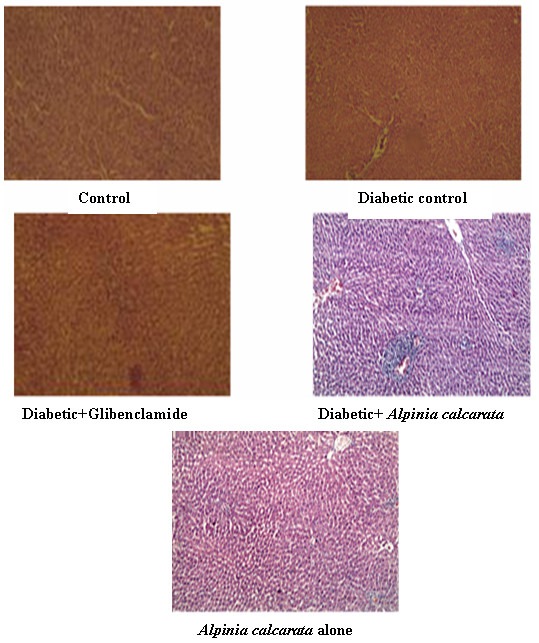
Histopathological examination of liver (100x magnification).

Histopathology of the liver of control animals showed normal hepatic structure. In diabetic control rats, liver sections showed sinusoidal dilation, feathery degeneration and necrosis. In diabetic animals treated with glibenclamide, liver sections maintained lobular architecture, mild sinusoidal dilation, and congestion. Liver of diabetic animals treated with ethanolic extract of *Alpinia calcarata* showed mild periportal aggregation of lymphocytes. The sinusoids appeared mildly congested and the central veins are appeared normal. There was no hepatocellular necrosis and no evidence of steatosis. In the case of liver sections of rats treated with *Alpinia calcarata* extract alone, the control veins, portal triads, zone 1, 2 and 3 hepatocytes and the sinusoidal spaces appear normal (Figure 2).

**Figure 3:**
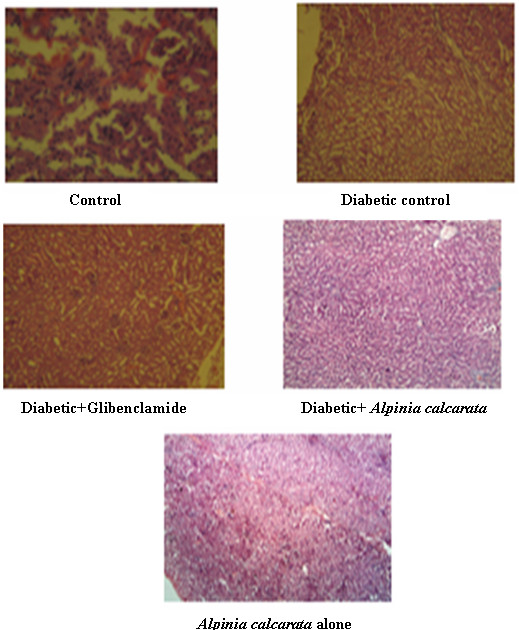
Histopathological examination of kidney (100x magnification).

Histopathology of the kidney (Figure 3) in control animals, showed normal histology. In diabetic control, kidney section showed severe tubular epithelial atrophy. In diabetic animal treated with glibenclamide, kidney sections maintained mild tubular epithelial atrophy. Kidney of diabetic animal treated with ethanolic extract of *Alpinia calcarata* showed mild tubular atrophy. The normal animal treated with plant extract of *Alpinia calcarata* alone showed normal histological structure.

**Figure 4:**
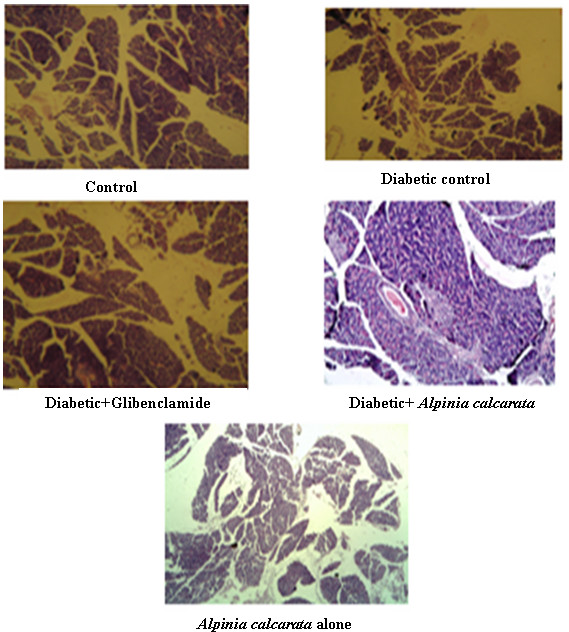
Histopathological examination of pancreas (100x magnification).

Histopathology of the pancreas (Figure 4) of control animals showed normal histology. In diabetic control rats, pancreas sections showed severe congestion of pancreatic parenchyma and hyperplasia of cells. In the case of diabetic animals treated with glibenclamide, pancreas sections maintained mild hyperplasia of islet cells. Pancreas of diabetic animal treated with ethanolic extract of *Alpinia calcarata showed* normal exocrine pancreas. The islet cells appeared normal and there was no inflammation or deposition of amyloid. A peripancreatic lymph node seen in histopathology which was also appeared normal. In case of the normal animals treated with ethanolic extract of *Alpinia calcarata*, studied showed exocrine pancreatic tissue, adipose tissue and a few islands of endocrine tissue.

## Conclusion

Thus the present study showed that the rhizome of *Alpinia calcarat*a possesses antidiabetic and antihyperlipidemic effects in streptozotocin induced diabetic rats. It can be concluded from the data that ethanolic extract rhizome extract supplementation is beneficial in controlling the blood glucose level, improves the lipid metabolism and prevents diabetic complications from lipid peroxidation and antioxidant systems in experimental diabetic rats. This could be useful for prevention or early treatment of diabetic disorders.

## Competing interest

We, the authors declare that there is no competing interest.

## Authors’ contribution

‘RR’ and ‘KM’ designed the study. ‘RR’ develop the protocol and wrote the first and second drafts of the manuscript. ‘RR’ and KM’ performed the experimental works. ‘NR’ ‘GD’ and ‘DK’ involved in the collection of literature, interpret the results and performed the statistical analysis. All authors read and approved the final manuscript.
